# d-Amino Acids and pLG72 in Alzheimer’s Disease and Schizophrenia

**DOI:** 10.3390/ijms222010917

**Published:** 2021-10-09

**Authors:** Yu-Jung Cheng, Chieh-Hsin Lin, Hsien-Yuan Lane

**Affiliations:** 1 Department of Physical Therapy, Graduate Institute of Rehabilitation Science, China Medical University, Taichung 40402, Taiwan; chengyu@mail.cmu.edu.tw; 2 Department of Rehabilitation, China Medical University Hospital, Taichung 40402, Taiwan; 3 Institute of Clinical Medical Science, China Medical University, Taichung 40402, Taiwan; 4 Graduate Institute of Biomedical Sciences, China Medical University, Taichung 40402, Taiwan; 5 Department of Psychiatry, Kaohsiung Chang Gung Memorial Hospital, Chang Gung University College of Medicine, Kaohsiung 83301, Taiwan; 6 School of Medicine, Chang Gung University, Taoyuan 33302, Taiwan; 7 Department of Psychiatry & Brain Disease Research Center, China Medical University Hospital, Taichung 40402, Taiwan; 8 Department of Psychology, College of Medical and Health Sciences, Asia University, Taichung 41354, Taiwan

**Keywords:** Alzheimer’s disease, schizophrenia, d-amino acids, pLG72

## Abstract

Numerous studies over the last several years have shown that d-amino acids, especially d-serine, have been related to brain and neurological disorders. Acknowledged neurological functions of d-amino acids include neurotransmission and learning and memory functions through modulating *N*-methyl-d-aspartate type glutamate receptors (NMDARs). Aberrant d-amino acids level and polymorphisms of genes related to d-amino acids metabolism are associated with neurodegenerative brain conditions. This review summarizes the roles of d-amino acids and pLG72, also known as d-amino acid oxidase activator, on two neurodegenerative disorders, schizophrenia and Alzheimer’s disease (AD). The scope includes the changes in d-amino acids levels, gene polymorphisms of *G72* genomics, and the role of pLG72 on NMDARs and mitochondria in schizophrenia and AD. The clinical diagnostic value of d-amino acids and pLG72 and the therapeutic importance are also reviewed.

## 1. Introduction

d-amino acids, in which the stereogenic carbon alpha of the amino group has a d-configuration, are agonists or co-agonists of *N*-methyl-d-aspartate type glutamate receptors (NMDARs). NMDARs are crucial in activity-dependent synaptic strength and structural changes related to long-term synaptic plasticity [1]. Thus, the levels of d-amino acids and their synthesis and degradation are linked to cognitive impairment, which is the core feature of schizophrenia and Alzheimer’s disease (AD). Indeed, studies show that the enzymes involved in d-amino acids metabolism correlate with AD and schizophrenia [2,3].

Schizophrenia and AD, the two most common neurological disorders, cause similar substantial cognitive and behavioral impairment. Although the pathophysiology of AD and schizophrenia are distinct, dysfunction of NMDARs transmission plays a critical role in their pathophysiology. An increasing number of studies focus on factors that regulate NMDAR activity in cellular and animal models of these two diseases. For instance, pLG72, also known as d-amino acid oxidase activator (DAOA) and able to modulate d-amino acid metabolism, is thought to be highly related to AD and schizophrenia pathogenesis. Polymorphisms in the *G72* gene have been connected to behavioral and psychological symptoms in patients with AD [4] and schizophrenia [5]. In addition to regulating d-amino acid levels, pLG72, localized in the mitochondria, is involved in the cellular oxidative stress defense system [6]. These pieces of evidence indicate that pLG72 plays a vital role in AD and schizophrenia and that targeting pLG72 could be a potential strategy for AD and schizophrenia treatment.

To elucidate the role of d-amino acids and pLG72 in AD, we first briefly summarize the metabolic pathways of d-amino acids and the changes of d-amino acid levels in AD and schizophrenia. Second, we discuss the known genetic variants in the *G72* gene and the changes in pLG72 correlation in schizophrenia and AD. Third, we describe the possibility of therapeutically targeting the d-amino acids metabolism in schizophrenia and AD.

## 2. d-Amino Acids, d-Amino Acids Metabolism, and NMDAR

### 2.1. Source of *d*-Amino Acids

All amino acids except for glycine are stereoisomers. It is commonly assumed that l-amino acids are predominant in living organisms and that d-forms are primarily found in microorganisms and bacteria. Recent studies have shown that d-amino acids are also present in mammals. The sources of d-amino acids include microbial production, ingestion, and racemization. Endogenous d-amino acids are produced through racemization from their corresponding antipodes by racemases [7]. Hence, amino acid racemases play a crucial role in d-amino acids metabolism. Serine racemase (SR) and aspartate racemase are found in mammals. However, only serine racemases have been detected in human tissues [8,9,10]. Wolosker et al. cloned and purified SR, and they also found that SR is highly selective for l-serine [11]. In addition to d-serine synthesis, SR also participates in d-aspartate biosynthesis [12].

**Figure 1:**
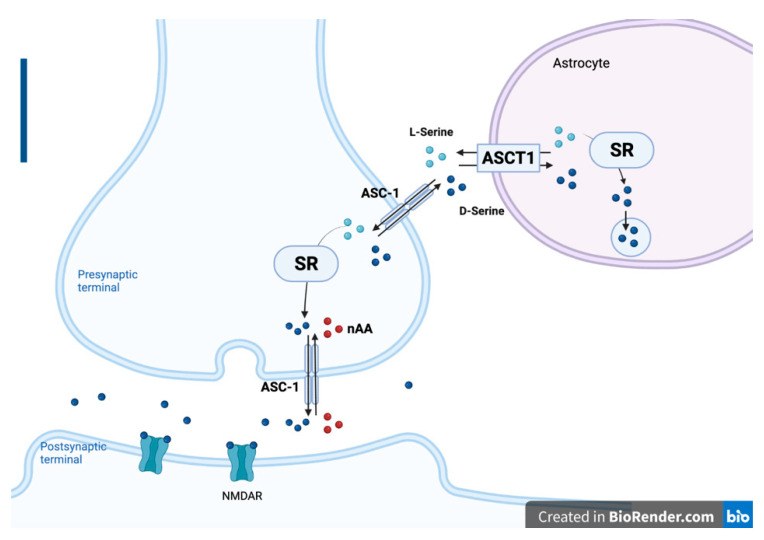
Illustration of the serine shuttle model. d-serine is converted from l-serine by serine racemase (SR) in astrocytes and presynaptic neurons. In astrocytes, d-serine also accumulates in vesicles. d-serine can be shuttled from neurons to astrocytes by alanine-serine-cysteine-1 transporter (Asc-1) and alanine, serine, cysteine, and threonine exchangers (ASCT1), whereas l-serine is shuttled from astrocytes to neurons by the same transporters in the opposite direction. The excessive d-serine in the synaptic cleft is removed by Asc-1 (created with BioRender.com accessed on 25 September 2021).

SR can convert d-serine from l-serine in the brain. Early studies have indicated that there are concentrations of d-serine and SR in astrocytes [11,13] and that this glia-derived d-serine modulates NMDA receptor activity and synaptic memory [14]. More recent studies have shown high amounts of SR and d-serine in primary neuronal cultures and neurons in vivo [15,16]. l-serine, produced in astrocytes, can be released by alanine, serine, cysteine, and threonine exchangers (ASCT1, SLC1a4) in exchange for d-serine and other amino acid substrates [17]. l-serine is further transported to neuronal cells by alanine-serine-cysteine-1 transporter (Asc-1, SLC7A10), which contributes to the uptake of l-serine in neurons [18]. The l-serine that is shuttled to neurons is converted to d-serine by SR. In the opposite direction, d-serine is released from neurons by an antiporter Asc-1 [19]. d-serine, taken up by astrocytes through the ASCT1 transporter, accumulates in the glial vesicles [17,20]. A serine shuttle model has been proposed to summarize the activity of l-serine, d-serine, Asc-1, and ASCT1 transporter [21] (Figure 1). In addition, primary astrocytes from ASCT1-KO mice, but not from ASCT2-KO mice, exhibited a reduced ability to uptake d-serine, l-serine, l-alanine, l-threonine, and glycine [17].

d-amino acids can be found extensively in foods and beverages, such as vegetables, fruits, wine, milk, beer, and fermented foods [22]. These acids are also generated during food processing; for example, bacteria and yeast produce d-amino acids during fermentation [23]. A study on vinegar found that the total d-amino acid level in lactic-fermented tomato vinegar was high, and lactic acid bacteria have a greater ability to produce d-amino acids than do yeast or acetic acid bacteria [24]. The alteration of d-amino acid concentration might depend on specific bacteria during red and white wine fermentation [25].

In addition to d-aspartate and d-serine, which mammals can synthesize endogenously, several d-amino acids such as d-alanine, d-glutamate, d-aspartate, d-serine, and d-proline, can be absorbed from gut bacteria [26,27]. Interestingly, d-amino acids, d-amino acids oxidase (DAAO), and microbiota on the epithelial surface of the small intestine cooperate and maintain the homeostasis of murine mucosa immunity [26]. The brain–gut–microbiota axis indicates a possible linkage between the bacteria in the intestine and the development of neurological disorders, such as Parkinson’s disease, AD, multiple sclerosis, schizophrenia, and autism [28,29,30]. By analyzing fecal samples from patients with AD and matched cognitively healthy controls, Zhung et al. found that gut microbiota composition was different between the two groups [31]. Similar to AD, studies showed a smaller subset of bacteria is different in schizophrenia patients compared with the control group [32]. Thus, one possibility is that gut bacteria from different microbiota environments generate different d-amino acids involved in the pathogenesis of AD and schizophrenia.

### 2.2. Elimination of *d*-Amino Acids and *d*-Amino Acid Oxidase

The flavin enzyme DAAO is found mainly in the kidney, liver, and brain of mammals [33,34]. By a reduction of its flavin adenine dinucleotide (FAD) coenzyme, DAAO oxidizes d-amino acids and produces imino acid. Afterward, the hydrolysis of imino acid causes the production of α-keto acid [35]. Mammalian DAAO enzyme activity has been identified in the rat brain, as well as in bovine and human nerve tissue [36,37]. DAAO activity is rare in the forebrain but high in the lower brain [38], and the localization of DAAO is inversely correlated with the presence of d-serine [39]. In addition, DAAO enzyme activity and mRNA are increased in the cerebellum after birth [40,41]. Cellular expression of DAAO is intense in glial cells, and histochemical studies observed intense DAAO activity in astrocytes, including those in Bergmann glial cells [36,38,42]. Immunohistochemistry staining and in situ hybridization have identified DAAO in glial cells in the brains of rats and humans [40,43,44].

After the administration of d-serine orally, plasma d-serine levels were significantly higher in DAAO knockout mice than in wild-type mice [45]. Using mutant ddY/DAAO– mice lacking DAAO activity, Hashimoto et al. found that d-serine levels in the serum and cerebellum of mutant mice were much higher than in normal mice [46]. These findings indicate that DAAO is responsible for metabolizing d-serine. In addition, the catabolic enzyme d-aspartate oxidase (DASPO or DDO) regulates d-aspartate [47]. Mice lacking DDO had increased amounts of both d-aspartate and NMDA in all tissues [48]. Errico et al. investigated whether chronic treatment with d-aspartate and DDO gene deletion may trigger plastic modifications of neuronal cytoarchitecture in the prefrontal cortex and CA1 subfield on the hippocampus in mice [49]. Taken together, DAAO and DDO are both responsible for catabolizing d-serine and d-aspartate, which serve as co-agonists of NMDA receptors (NMDARs); this implies that DAAO and DDO are involved in neuronal function.

### 2.3. *d*-Amino Acids and NMDAR Function

Among all d-amino acids, d-aspartic acid, d-glutamate, d-alanine, and d-serine can modulate NMDARs directly by binding to glutamate or glycine binding sites [50]. After removing the Mg^2+^ block and binding two agonists, glutamate and glycine, the NMDARs are activated and induce Na^+^, K^+^, and Ca^2+^ currents [51,52,53]. Several studies indicate that Ca^2+^ overload plays an essential role in NMDARs-mediated neurotoxicity [54,55,56]. The overactivation of extrasynaptic GluN2A-containing NMDARs might trigger glutamate excitotoxicity, which is correlated with cognitive decline and neurodegeneration in patients with AD. As NMDARs antagonist memantine reduces glutamate excitotoxicity, it provides both symptomatological and neuroprotective benefits in moderate to severe AD [57,58,59,60]. However, synaptic NMDARs are critical for neuronal survival [61,62]. Thus, the preservation of adequate NMDAR activity helps maintain neuronal survival when treating AD with NMDAR antagonists. Because NMDARs-mediated synaptic dysfunction affected by d-amino acids is a pathological change associated with AD, researchers have increasingly investigated the association between d-amino acids and AD. More and more studies focus on the alteration of d-amino acids and their metabolic pathways in AD.

### 2.4. Changes of *d*-Amino Acids, DAAO, and SR Levels in AD Patients

The changes in d-amino acid levels in patients with AD are well-studied; however, the results of these studies are controversial. It might have resulted from small sample size and different analytical methods. The methods to detect amino acid levels in some earlier articles cannot distinguish between free d-amino acids and d-amino acids derived from digested proteins [2]. Since d-serine is the main co-agonist of the NMDA receptor in the frontal brain [63,64,65], many studies have investigated the possible alteration of d-serine levels in AD. Madeira et al. observed that d-serine levels in the cerebrospinal fluid (CSF) of patients with a probable AD diagnosis were 5 times higher than CSF d-serine levels of controls [66]. In the same study, d-serine levels in postmortem hippocampal and cortical samples from AD patients were increased [66]. In a study on 376 individuals, Lin et al. found that d-serine levels and the d-serine to total serine ratio were significantly higher in patients with AD [67]. Another study from the same research team also revealed a significantly higher level of serum d-serine in patients with AD [68]. Compared with age-matched healthy controls, the serum d-serine levels and the d-serine to total serine ratios were significantly higher in individuals with AD progression [69]. However, several studies have revealed no statistically significant differences in the free d-serine levels in the frontal or parietal cortex between patients with AD and controls [70,71,72,73]. Nuzzo et al. analyzed the d-serine concentrations in the blood serum and CSF of patients representing the whole clinical spectrum of AD. They found that there was no alteration of d-serine levels in the blood serum or CSF. Moreover, there was no identified correlation between serum or CSF d-serine concentration and Mini-Mental State Examination scores [73]. Biemans et al. analyzed d-serine levels in the CSF of individuals with or without AD and found no notable difference between the two groups [74]. A systematic review and meta-analysis that included seven trials demonstrated that serum and CSF d-serine levels were significantly higher in patients with AD than in controls [75]. Thus, d-serine levels are a potential biomarker for detecting AD.

The concentration of d-aspartate, another NMDA receptor co-agonist, was also quantified in the brains of individuals with and without AD. Aged-related accumulation of d-aspartate was found in the white matter but not the gray matter of normal brains [76]. d-aspartate levels were more than twice as high in the white matter of normal brains than in the white matter of AD brains, whereas the d-alanine concentration was more than twice as high in AD gray matter than in normal gray matter [77]. Using a new procedure to hydrolyze proteins without provoking racemization of amino acids, Fisher and colleagues found that d-aspartate concentration in both the gray matter and white matter of AD brains was significantly higher than that in healthy brains [78]. Higher levels of d-aspartic acid in the CSF of patients with AD have also been found compared with the ventricular CSF of healthy controls [79,80]. The level of d-alanine in the white and gray matter of AD brains was higher than in healthy brains, whereas total alanine was significantly lower in the gray matter of AD brains compared with healthy brains [81].

Research also suggested that there is a correlation between the changes in the levels of d-glutamate and AD. Cognitive decline in patients with AD is strongly correlated with decreased d-glutamate levels in blood [68,82,83]. Furthermore, lower hippocampal glutamate concentrations were found in patients with mild cognitive impairment (MCI) and AD compared with cognitively healthy older adult controls [84]. Vijayakumari and colleagues enrolled 15 patients with MCI and healthy controls and used functional magnetic resonance imaging (fMRI) to evaluate glutamate responses during working memory tasks. The results showed a significant increase in glutamate response during a working memory task in healthy participants but no observed significant changes in glutamate response in patients with MCI [85]. These studies indicate that dysregulated d-glutamate levels and glutamatergic neurotransmission may be associated with cognitive function in patients with AD or MCI.

Blood DAAO level could serve as a potential surrogate biomarker for AD. Our previous study examined serum DAAO levels and cognitive function in patients with MCI, mild AD, moderate to severe AD, and healthy older adults. The peripheral DAAO levels increased with the severity of cognitive deficits, and the Clinical Dementia Rating Scale (CDR) score was significantly associated with the DAAO level [68]. A similar increase in DAAO in the blood of patients with post-stroke dementia was noted, and the plasma DAAO levels were independently higher in subjects with dementia than subjects without dementia [86]. Treating with DAAO inhibitors, such as sodium benzoate, can ameliorate cognitive decline in patients with AD [87,88], indicating that over-activated DAAO plays a vital role in AD. In addition, several studies have demonstrated a direct association between SR and neurodegenerative diseases. Wu et al. observed that Aβ-peptide increased SR expression and d-serine concentration in cultured microglia. Compared with age-matched controls, the levels of SR mRNA were higher in the hippocampus of patients with AD [89]. In SR knockout mice, d-serine levels were reduced by approximately 90% in the forebrain, and NMDA- and Aβ- peptide-induced neurotoxicity was also significantly attenuated. These results suggest that SR is the primary enzyme for d-serine production, and d-serine may be involved in NMDA receptor-mediated neurotoxicity [90]. SR knockout mice exhibited impaired spatial memory and anxiety due to alteration in glutamatergic neurotransmission [91]. However, a recent study revealed that SR knockout mice, which had a weaker long-term potentiation (LTP) and a smaller increase in NMDA receptor potentials, had no deficits in spatial learning, reference memory, or cognitive flexibility. The significant increase in glycine levels [92] may explain the preservation of memory ability.

### 2.5. *d*-Amino Acids and NMDAR Function in Schizophrenia

In addition to the dopamine hypothesis, which is the main point on the pathophysiology of schizophrenia, more studies have shown that the dysfunction of NMDARs contributes to schizophrenia [93,94,95]. A large body of evidence has shown that there are altered d-serine levels in patients with schizophrenia. Bendikov et al. monitored the d-serine levels in CSF and SR expression in postmortem brains of schizophrenic patients. The study showed that the d-serine levels and d/l-serine ratio in CSF decreased by 25%, and SR protein levels in the frontal cortex and hippocampal in postmortem brain of schizophrenic patients were also reduced [96]. Hashimoto et al. reported a reduced d-serine to total serine ratio in the CSF of drug-naïve schizophrenic patients [97]. However, another study had revealed no statistically significant differences in the d-serine levels in CSF between patients with schizophrenia and control [98]. In the same study, the levels of l-serine, l-glutamine, and l-glutamate in CSF were unchanged [96]. Other studies also showed that serum d-serine levels in the patients with schizophrenia were significantly lower than in the healthy controls [99,100]. A systematic review summarized six studies, and results from the meta-analysis showed that patients with schizophrenia had lower serum d-serine levels than healthy controls [101]. In summary, generally increased d-serine level was observed in AD and decreased d-serine was reported in schizophrenia patients (see Table 1).

Since Aβ can induce SR expression and d-serine release in microglia [89], it is not surprising that the d-serine level increases along with AD progression. In addition, the racemization of protein-bound amino acids is significant in protein aging and aggregation, which contributes to neurodegeneration [102]. The Aβ1-40, racemized at Ser26, is soluble and susceptible to proteolysis, which converts into toxic fragments [103], and this racemized amyloid-β is associated with AD pathogenesis [104]. Hence, the d-serine from proteolytic Aβ might also contribute to the elevated d-serine levels in AD patients. Different from AD, there is no evidence showing that Aβ levels are different in patients with schizophrenia than in controls, and the excessiveness of Aβ is not associated with cognitive impairment in schizophrenia [105]. Thus, the differential pattern of d-serine levels between AD and schizophrenia might have resulted from the accumulation and proteolysis of Aβ.

Interestingly, a study monitoring changes in glycine, l-serine, and d-serine levels in the plasma of schizophrenic patients during treatment showed that there was a significant increase in d-serine levels along with an improvement in clinical symptoms [106]. Thus, d-serine levels are a potential “clinical status” biomarker in patients with acute schizophrenia. Moreover, clinical trials have shown that d-serine treatment significantly improves positive, negative, and cognitive symptoms in patients with schizophrenia and bipolar disorder [107]. In addition, several studies report increased mRNA, protein, or enzyme activity of DAAO in subjects with schizophrenia [40,108,109,110]. Verral et al. analyzed different brain regions in autopsies of schizophrenic patients, and they found DAAO increased in the cerebellum, whereas SR mRNA increased in the dorsolateral prefrontal cortex [40]. The distinct expression pattern in different brain regions of schizophrenic patients implies that the regulation of d-serine by DAAO/SR needs further investigation.

The concentration of d-aspartate, another NMDA receptor co-agonist, was found reduced in the prefrontal cortex and striatum in schizophrenic patients [111]. The concentration of d-aspartate is markedly high in the embryonic brain and then rapidly decreases after birth due to DDO [112,113,114]. In DDO knockout mice, increased d-aspartate attenuates phencyclidine-induced schizophrenia-like behaviors [115]. The same group reported that d-aspartate not only can increase in vivo NMDAR activity but also can attenuate schizophrenia-like symptoms induced by amphetamine and MK-801 [116]. Consistent with the animal model results, there is a link between low d-aspartate levels and enhanced DDO activity in the dorsolateral prefrontal cortex of schizophrenic patients [117]. However, exposure to excessive d-aspartate in DDO knockout mice facilitated age-dependent brain neurodegeneration processes, which indicated the excitotoxicity of NMDA receptors [118]. Keller et al. analyzed the mRNA levels and DNA methylation status of SR, DAAO, *G72*, and DDO in postmortem brain samples from patients with schizophrenia and controls [119]. They found that the DDO methylation and expression were lower in the cerebellum compared with the hippocampus and dorsolateral prefrontal cortex. Although there was no difference between healthy controls and schizophrenia patients, the finding from DNA methylation is consistent with the DDO gene activity in the cerebellum. Moreover, a single nucleotide polymorphism (SNP) rs3757351 on the DDO gene is also associated with reduced DDO expression in in vivo prefrontal phenotypes relevant to schizophrenia and greater prefrontal gray matter volume [49]. These results implicated the dysregulation of d-aspartate as perhaps playing an important role in schizophrenia [120].

Glutamate is the primary excitatory neurotransmitter, and glutamatergic theory is one of the most influential hypotheses in schizophrenia. Some studies found serum glutamate level was significantly higher in schizophrenia patients [121,122,123]. However, the plasma glutamate level is not correlated with its level in the brain because glutamate is synthesized too in the central nervous system [124,125]. Although d-glutamate is primarily degraded by DDO in eukaryotic organisms [126], d-aspartate, but not d-glutamate, was significantly increased in DDO knockout mice brain, which suggested that DDO mainly catalyzes d-aspartate, not d-glutamate [127]. Ariyoshi and co-authors identified a novel mammalian mitochondrial protein 9030617O03Rik, a d-glutamate cyclase that converts d-glutamate to 5-oxo-d-proline [128]. Unlike AD, there is no evidence showing a correlation between d-glutamate cyclase and schizophrenia.

As mentioned, NMDARs hypofunction contributes to schizophrenia, and d-serine is the primary NMDAR co-agonist, and it is synthesized by SR. It is not surprising that there is an association between SR activity and schizophrenia pathophysiology. Labrie et al. induced SR gene mutation and generated mice with low SR activity and s-serine level. These mice displayed behaviors relevant to schizophrenia, including impairments in prepulse inhibition, sociability, and spatial discrimination [129]. Basu and co-authors generated SR knockout mice, but SR–/– mice exhibited intact prepulse inhibition (PPI) response, which was impaired in schizophrenic patients at the acute stage [91]. Although d-serine levels were reduced by approximately 90% in SR knockout mice, the intact PPI response might have resulted from other NMDARs co-agonist glycine. Regardless of the behavioral phenotype, SR knockout mice showed some pathologic features of schizophrenia, such as loss of cortical gray matter, reduction of cortical glutamatergic synapses, downregulation of parvalbumin-positive cortical GABAergic neurons, and cognitive development impairments [130,131,132]. Recent studies show that dysregulation of SR/Disrupted-In-Schizophrenia-1 (DISC1) also contributes to schizophrenia. DISC1 binds to SR, and disruption of SR/DISC1 complex caused schizophrenia-like behavior through d-serine depletion [133,134]. Moreover, few SR genetic variants associated with schizophrenia have been identified in humans [129,135]. Later, a genome-wide association study of 36,989 patients with schizophrenia and 113,075 controls also showed the SNP rs4523957 of the SR gene is one of the gene variants associated with schizophrenia [136], and rs4523957 may be associated with some phenotypes of schizophrenia in the Han Chinese population [137].

## 3. d-Amino Acids Oxidase Activator, *G72*

### 3.1. Biological Function of G72 and the Possible Role of G72 in AD

*G72* is a primate-specific gene located in the 13q33.2 chromosomal region and encodes a protein with 153 amino acid residues. In 2002, Chumakov et al. first described strong associations between schizophrenia and SNPs of *G72* [138]. This study also reveals that the pLG72 protein binds to DAAO through the yeast two-hybrid technique, and the in vitro result shows that pLG72 protein can activate DAAO. Reverse transcription-polymerase chain reaction (RT-PCR) and real-time PCR results show that *G72* expression is only detected in human testis and brain [139]. In *G72* transgenic mice, expression of pLG72 in the cerebellum, hippocampus, cortex in the brain, heart, testis, and spleen in peripheral tissue was found [140,141]. Transgenic mice with an overexpressed human *G72/G30* genomic region show behavioral phenotypes related to schizophrenia and depression [141], which indicates that the *G72* gene does play a role in modulating behaviors.

There are three possible mechanisms of how pLG72 modulates behaviors. The first one is through pLG72–DAAO–NMDAR; second, it can modulate mitochondrial function; and last, it can induce oxidative stress. Although Chumakov et al. proposed that G72 can bind DAAO and serve as a DAAO activator, the effects of pLG72 in regulating DAAO activity are still inconclusive. According to a detailed functional analysis, the inhibitory effects of pLG72 on DAAO have been reviewed comprehensively [6]. The protein–protein interaction of DAAO and pLG72 was confirmed at the in vitro and cellular level by several different groups [44,138,142,143,144]. Yeast two-hybrid experiments demonstrated that pLG72 interacts with DAAO in vitro and that there was an increase in the activity of DAAO in the presence of pLG72 [138,145]. Immunohistochemical staining and immunoprecipitation showed that there was an expression and interaction of DAAO and pLG72 in the human cortex [44]. Nevertheless, co-expression of pLG72 with DAAO in glioblastoma cells abolishes the effects of DAAO, which indicates that pLG72 may also act as a repressor of DAAO [44]. The same group reported that pLG72 briefly interacted with DAAO in the cytosol and negatively affected the half-life of DAAO [146]. Another group also failed to repeat the DAAO activator function of pLG72 in Gos7 and U251 glioblastoma cells [147]. Sacchi and co-authors tested the DAAO modulation functions of wild-type pLG72, R30K variant, and K62E variant. They found that pLG72 variants, including wild-type pLG72, actually inhibit human DAAO, which leads to increasing d/d+l-serine levels [142]. Through pLG72-directed DAAO activity assay, Terry-Lorenzo et al. found that pLG72 can dose-dependently inhibit DAAO [148]. Testing in various human cell lines suggested that the effects of pLG72 on DAAO activity are cell type-dependent [144]. As mentioned, DAAO modulates d-serine levels, which regulate NMDAR biological function. Thus, the interaction between pLG72 and DAAO links abnormal d-serine levels and NMDAR dysfunction-related neurological disorders, including AD [2].

**Figure 2:**
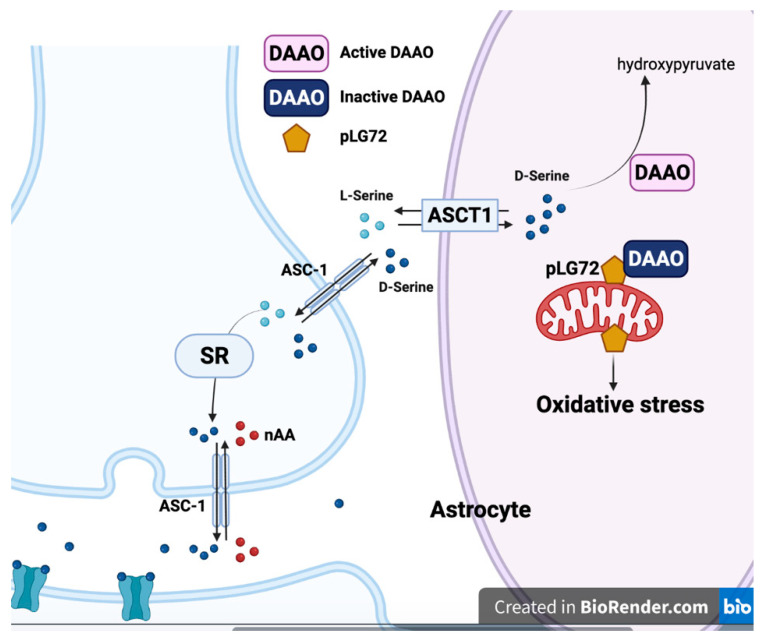
The DAAO–pLG72 interaction in d-serine degradation and mitochondria stress. d-serine is degraded by DAAO in astrocytes. pLG72, when on the mitochondrial outer membrane, can bind and inhibit DAAO. Overexpressed pLG72 on mitochondria also induces mitochondria fragmentation and oxidative stress (created with BioRender.com accessed on 25 September 2021).

In addition to modulating DAAO activity, there was also a report regarding *G72* affecting mitochondrial function. The *N*-terminal of pLG72 contains a mitochondrial translocation sequence, and immunostaining results show that pLG72 is mainly localized in mitochondria [147]. Increased pLG72 proteins induced mitochondrial fragmentation without inducing apoptosis in the Gos7 cell line and primary neurons. Moreover, transfection with pLG72 in primary hippocampal neurons changed mitochondrial morphology and dendritic branching [147]. Interestingly, overexpression of pLG72 affected several mitochondrial-related gene expressions and increased reactive oxygen species (ROS) in U87 glioblastoma cells [149]. pLG72 transgenic mice show schizophrenia-relevant behaviors, mitochondrial dysfunction, and higher ROS production [150]. Since ROS, generated by mitochondria, participates in many neurodegenerative diseases, there has been a link between pLG72-associated ROS production and AD. Treating with antioxidant N-acetyl cysteine can improve the cognitive deficits of pLG72 transgenic mice [150]. Through the yeast-two hybrid system, the same group reported that mitochondrial methionine sulfoxide S reductase B2 (MSRB2) is the binding partner of pLG72 [151]. Increasing pLG72 protein expression induces mitochondrial oxidative stress by altering MSRB2 function. In addition to MSRB2, pLG72 was reported to bind Flavin mononucleotide (FMN) and modulate FMN-containing oxidoreductase activity in the respiratory complex I [44,142]. Interestingly, MSRB2-mediated antioxidative activity can reduce amyloidogenesis and Tau phosphorylation in the AD mice model [152]. These results imply that the role of pLG72 on mitochondria dysfunction may enhance AD pathogenesis (Figure 2).

### 3.2. Role of G72 in Schizophrenia

The association of neurological disorders and *G72* gene variations was first discovered in schizophrenia and psychiatric conditions [153,154,155,156]. In the paper reporting the discovery of the *G72* gene, Chumakov et al. suggested a possible link between pLG72 and schizophrenia due to its DAAO modulation function [138]. Although Sacchi et al. showed contradicting results to Chumakov’s finding and demonstrated that pLG72 acts as a negative effector of human DAAO, they proposed that increased DAAO and decreased pLG72 expression might lower d-serine concentration and be involved in schizophrenia susceptibility [44]. Ishiwata’s group showed a significant positive correlation between plasma *G72* levels and positive symptoms of schizophrenia [157]. However, a tendency toward overexpression of *G72* mRNA and protein in the brains of schizophrenic patients compared with healthy controls was observed [158]. Similar to the brain findings, a significant elevation of *G72* protein in the plasma of schizophrenic patients was detected compared with healthy controls [159]. While the pLG72 R30K, the best-known SNP rs2391191 in *G72*, has a shorter half-life than wild-type pLG72, the overexpressed pLG72 in schizophrenia patients might be due to the compensatory result.

Plenty of studies have analyzed genetic variants and tried to link these markers with schizophrenia. SNP-based studies showed that several *G72* gene variants were associated with schizophrenia in different populations [138,158,160,161,162,163,164,165]. However, some SNPs (rs3916965, rs1341402, rs2391191, rs778293, and rs3918342) show controversial associations in different studies, possibly resulting from different populations [139]. Lin et al. analyzed the *G72* SNPs and *G72* protein levels from schizophrenia patients and healthy controls with leveraging computational artificial intelligence and machine learning. They found *G72* rs1421292 plus *G72* protein seemed to be the best model for schizophrenia susceptibility [5]. SNP rs2391191 and rs9558562 induce R30K and K62E substitution, R30K pLG72 has a shorter half-life, and K62E has higher DAAO inhibition than wild type [6]. Moreover, schizophrenic patients who carried the rs2391191 variant appeared to have a significantly thinner cortex, which indicated the pathophysiological role of pLG72 in schizophrenia [166]. Hall et al. analyzed the brain activation with functional magnetic resonance imaging in subjects carrying two *G72* SNPS, rs3918342 and rs1421292. Their results showed differences in the activation of the left hippocampus and parahippocampus between *G72* genotype groups, which indicated that genetic variation of *G72* may affect hippocampal complex and prefrontal cortex function [167].

### 3.3. Association of G72 with AD

Similar to schizophrenia, genetic variation of the *G72* gene was found to be connected with AD. After screening 185 AD patients for SNP, one SNP—rs2153674—was associated with more frequent and severe delusions assessed with the Neuropsychiatric Inventory [4]. Arcos-Burgos and his team analyzed the world’s largest multigenerational pedigree with early onset of AD, carrying the PSEN1 p.Glu280Ala mutation. With pooling/bootstrap-based genome-wide association studies (pbGWAS), they first reported a novel locus including *G72* (rs778296) that is significantly associated with the early onset of AD [168]. Later they found that an exonic missense mutation of *G72* (rs2391191) was also significantly associated with the age of onset by whole-exome genotyping of sixty individuals [169]. In addition, an association between *G72* gene variations and some AD- or dementia-related psychological disorders, including panic disorder [170], bipolar disorder, [156], persecutory delusions [171], and frontal lobe volume changes, was observed [172]. Results of GWAS have shown that AD and schizophrenia share similar physical phenotypes through standard GWAS signals [173] and that *G72* provides a good example of the links between these two diseases.

For protein expression level, a postmortem study showed that pLG72 protein expression levels at different ages was statistically significantly different in the brainstem [174]. Lin et al. first showed that the pLG72 level in plasma is about 2-fold higher in schizophrenic patients than in control subjects [159]. The same group further measured the pLG72 protein levels in the plasma of 376 participants who evaluated the severity of cognitive deficit through the Clinical Dementia Rating Scale. In patients with MCI, mild and moderate AD, the levels of pLG72 were increased compared with healthy subjects. However, pLG72 levels in severe AD were not significantly different [67]. These findings suggest that pLG72 may not be suitable as a biomarker for late AD. Moreover, the d-serine level and d- to total serine ratio in MCI and AD patients were significantly higher than in the healthy control. These findings suggest that pLG72-mediated DAAO modulation might contribute to NMDAR dysfunction and AD progression.

Several studies that analyzed *G72* gene variants showed a genetic association between AD and schizophrenia risk. Table 2 summarizes the genetic studies that had analyzed both disease groups. As shown in Table 2, one SNP, rs2391191, is related to an increase in the risk of AD and schizophrenia. According to the available results, there is an increased pLG72 expression in AD and schizophrenic patients (Table 3).

## 4. Therapeutic Targeting to d-Amino Acids Metabolic Pathways in AD and Schizophrenia

Given the contribution of NMDARs to learning and memory, it is unsurprising that medical researchers have attempted to improve cognitive function by enhancing NMDA receptor activity both in AD and schizophrenia. Although d-serine levels in brain tissues and the blood are not similar, therapeutic strategies targeting the d-amino acids metabolic pathway can improve cognition symptoms in both diseases. Both glycine and d-serine can bind to the glycine modulatory site of the NMDA receptor; however, d-serine has more significant potential. The median effective dose 50 (ED50) for d-serine activation of NMDARs is 3 to 4 times lower than glycine [178]. The extracellular concentration of free d-serine in the frontal cortex is sufficient to saturate the glycine binding site on the NMDA receptor in rodent brains [178]. After intravenous administration, d-serine had a higher uptake than had l-serine in rat brains [179]. A single systemic administration of d-serine resulted in the prolonged elevation of d-serine levels in the rat cortex and hippocampus [180], which are the affected brain regions in AD. In rats, an fMRI study found that intraperitoneal d-serine administration increased NMDA receptor activation in the hippocampus [181]. Moreover, d-serine treatment can enhance social memory in rats [182]. Both pre- and post-training treatment with d-serine improved recognition memory in mice [183]. These studies imply that increasing d-serine levels in patients with AD and schizophrenia might have positive effects.

As mentioned, the hypofunction of NMDARs plays a crucial role in schizophrenia, and d-serine is a potent co-agonist of NMDARs. Hence, it is not surprising that d-serine was tested to treat schizophrenia. Several studies have shown d-serine supplements did improve psychological symptoms of schizophrenia [107,184,185]. In addition to enhancing NMDARs activity by administrating d-serine directly, increasing endogenous d-serine concentration by inhibiting DAAO also resulted in therapeutic improvement. Sodium benzoate, a specific DAAO inhibitor, can increase d-serine level, reduce symptoms, and improve neurocognition in patients with chronic schizophrenia [186]. The same group showed that combined GlyT1 inhibitor sarcosine and sodium benzoate, not sarcosine, can improve cognition and global functioning of schizophrenic patients [187]. Moreover, similar beneficial effects of sodium benzoate on clozapine-resistant schizophrenic patients were also noted. Clozapine combined with sodium benzoate can improve the therapeutic effects of clozapine on symptomatology [188].

Due to the hypothesis in AD pathogenesis that excessive d-serine may induce NMDARs over-activation and neurotoxicity, there is no clinical trial testing the efficacy of d-serine on AD. However, d-serine treatment can enhance neurogenesis and the survival of newborn neurons [189], which implies that increasing d-serine levels might benefit AD. Indeed, reducing d-serine degradation by blocking DAAO activity, thereby enhancing NMDA receptor activity, can prevent neuronal cell death [190]. Sodium benzoate is a selective inhibitor of DAAO, which can effectively reduce DAAO-mediated reactions in a dose-dependent manner [191,192,193]. In a 5XFAD transgenic mouse model of AD, oral feeding of sodium benzoate suppressed the activation of p21rac, oxidative stress, neuronal apoptosis, glial activation, and Aβ deposits in the hippocampus [194]. In addition, several clinical trials have evaluated the efficacy of sodium benzoate in patients with MCI and AD. A randomized, double-blind, placebo-controlled trial of patients with amnestic MCI and mild AD showed that 24 weeks of daily treatment with sodium benzoate improved cognitive and overall function [87]. Another double-blind, 6-week trial demonstrated that high-dose sodium benzoate treatment did not improve behavioral and psychological symptoms of dementia (BPSD) [195]. However, after improving the treatment with a precision medicine approach, sodium benzoate reduced DAAO levels and attenuated d-serine declines in patients with BPSD. Moreover, there is a correlation between cognitive improvement after sodium benzoate treatment and decreased DAAO levels [88]. These studies indicate that sodium benzoate may have the potential to improve cognitive function in early-phase AD. Notably, a second analysis of the same cohort showed that sodium benzoate treatment was more effective in women than in men for improving cognitive function [196], implying that sex hormones may play a role.

As mentioned in the previous section, serum d-serine levels in the early phases of AD can be a biomarker for clinical status. Since increased d-amino acids levels might have resulted from Aβ stimulation and digested products of Aβ peptides, the changes of d-amino acids levels after treatment are important. Aducanumab, the newly FDA-approved drug for AD, is designed to target oligomers and fibrils of Aβ [197]. The intravenous administration of aducanumab can reduce brain Aβ, which might be through increasing microglia-mediated phagocytosis of antibody–Aβ complexes [198]. Although clearance of racemized Aβ may reduce d-amino acids levels, the activation of microglia could also induce SR expression and release d-serine. Thus, it would be worth monitoring the changes in d-amino acid levels in patients treated with aducanumab.

## 5. Conclusions

Schizophrenia and AD, two neurological disorders, share psychiatric symptoms that result from NMDARs dysregulation. Among the regulators of NMDARs, more and more evidence shows that d-amino acids contribute to the pathogenesis of AD and schizophrenia. Thus, the genes/proteins that participate in d-amino acids metabolism serve as targets for treating schizophrenia and AD. pLG72 not only modulates DAAO but also affects mitochondria-related oxidative stress. The *G72* gene polymorphisms and pLG72 protein express similar patterns in schizophrenia and AD. Moreover, changes in d-amino acids, DAAO, and *G72* have been found in patients with schizophrenia and AD-related cognitive dysfunction, demonstrating their potential as therapeutic targets for both diseases. Indeed, the modulation of DAAO activity by DAAO inhibitors has shown effectiveness in several clinical trials on both diseases. However, more investigation into the efficacy and safety of treatments based on d-amino acid modulation in AD and schizophrenia is necessary.

## Figures and Tables

**Table 1:** Changes in d-serine levels in AD and schizophrenia samples.

Sample/Area	AD	Schizophrenia
Human autopsy/ Frontal cortex	↔ [71]	-
Postmortem human sample/ Superior frontal cortex	↔ [73]	-
Postmortem human sample/ Parietal cortex	↑ [66]	↔ [96]
Postmortem human sample/ Hippocampus	↑ [66]	-
Amyloid-β injected mice/ Hippocampus	↑ [66]	-
Human CSF	↔ [73], ↑ [66]	↓ [96], ↔ [97,98]
Human Blood (serum/plasma)	↔ [73] ↑ [2,67]	↓ [99,100]

↑: significantly increased, ↓ significantly decreased, ↔ no significant difference.

**Table 2:** Genetic association studies of *G72* genetic polymorphisms in AD and schizophrenia risk.

Polymorphisms	Position	Schizophrenia	AD	Global MAF [175]	MAF in Schizophrenia	MAF in AD
M15 rs2391191 (G>A/G>C)	chr13:104917447	+ [138,161,162,164]	+ [169]	0.370658 (A)	0.35(A) [138]	N/A
rs2153674 (C>A/C>G/C>T)	chr13:105478789	− [176]	+ [4]	0.47364 (C)	N/A	0.44 (C) [4]
M19 rs778294 (C>A/C>T)	chr13:104940236	+ [160] − [138,156,177]	− [4]	0.281331 (T)	0.253 (T) [160]	0.3 (A) [4]

+ represents significant association, and − represents no correlation.

**Table 3:** Changes in pLG72 levels in AD and schizophrenia.

Sample/Area	MCI	AD	Schizophrenia
Postmortem human sample/ dorsolateral prefrontal cortex	-	-	↑ [158]
Human CSF	-	-	↔ [157]
Human Blood (serum/plasma)	↑ [67]	↑ [67] (mild and moderate AD), ↓ [67] (severe AD)	↑ [159], ↔ [157]

↑: significantly increased, ↓ significantly decreased, ↔ no significant difference.

## Glossary

Asc-1	alanine-serine-cysteine-1 transporter
DISC1	Disrupted-In-Schizophrenia-1
FMN	flavin mononucleotide
fMRI	functional magnetic resonance imaging
NMDAR	*N*-methyl-d-aspartate type glutamate receptors
AD	Alzheimer’s disease
ASCT1	alanine, serine, cysteine, and threonine exchangers
BPSD	behavioral and psychological symptoms of dementia
CSF	cerebrospinal fluid
DAAO	d-amino acids oxidase
DDO	d-aspartate oxidase
ED50	effective dose 50
FAD	flavin adenine dinucleotide
MCI	mild cognitive impairment
MSRB2	mitochondrial methionine sulfoxide S reductase B2
pbGWAS	pooling/bootstrap-based genome-wide association studies
PPI	prepulse inhibition
ROS	reactive oxygen species
RT-PCR	reverse transcription-polymerase chain reaction
SNP	single nucleotide polymorphism
SR	serine racemase
